# Correlation Between Thyroid Function and Ambulatory Blood Pressure Monitoring

**DOI:** 10.3390/jcm14186580

**Published:** 2025-09-18

**Authors:** Nicia I. Profili, Edoardo Fiorillo, Michele Marongiu, Francesco Cucca, Alessandro P. Delitala

**Affiliations:** 1 Department of Medicine, Surgery, and Pharmacy, University of Sassari, 07100 Sassari, Italy; 2 Institute for Genetic and Biomedical Research, National Research Council, 08045 Lanusei, Italy; 3 Department of Biomedical Science, University of Sassari, 07100 Sassari, Italy

**Keywords:** hypertension, TSH, subclinical hyperthyroidism, diastolic blood pressure, male

## Abstract

**Background**: Blood pressure is associated with overt thyroid disorders, but the role of subclinical diseases is not clear, particularly when blood pressure is assessed at the clinical office. Ambulatory blood pressure monitoring over 24 h provides additional clinical information, which correlates with many cardiovascular endpoints. The aim of our work is to examine whether thyroid function is related to systolic and diastolic blood pressure assessed by ambulatory blood pressure monitoring. **Methods**: We enrolled 3277 subjects from the SardiNIA project. Thyroid function and ambulatory blood pressure monitoring were assessed in all the participants. **Results**: TSH was associated with average 24 h and daytime DBP in males but not in females, after adjusting for confounders (respectively, Coef −0.192 *p* = 0.025, and Coef. −0.021, *p* = 0.018). We found no association between TSH and DBP or SBP during nighttime. **Conclusions**: Low TSH in males is positively associated with high DBP. Further studies of underlying mechanisms will need to explore our findings.

## 1. Introduction

Thyroid hormones and the cardiovascular system are strictly connected. Overt and subclinical hypothyroidism are associated with accelerated atherosclerosis, dyslipidemia, and heart failure [1,2], while hyperthyroidism is associated with a higher incidence of all-cause mortality and atrial fibrillation [3,4].

Thyroid disorders are highly prevalent in the general population [5], and their identification, as well as their treatment, should be promptly performed to avoid specific unfavorable outcomes [6,7].

The prevalence of arterial hypertension is increased in both hypothyroidism and hyperthyroidism, although with different mechanisms. On the one hand, in overt hypothyroidism, impaired diastolic blood pressure (DBP) caused by increased systemic vascular resistance is more commonly found, and this effect may be reversed after levothyroxine treatment. On the other hand, patients with overt hyperthyroidism showed increased systolic blood pressure (SBP) due to decreased systemic vascular resistance. The role of subclinical disorders in this clinical context is not clear and evidence suggests a possible gender effect [8], particularly when blood pressure is assessed at the clinical office. Ambulatory blood pressure monitoring (ABPM) over 24 h provided additional clinical information compared to clinic blood pressure measurements, and further correlated with many cardiovascular endpoints. The lack of studies that analyzed the association between ABPM and thyroid disorders prompted us to examine whether thyroid function is related to systolic and diastolic blood pressure assessed by ABPM.

## 2. Material and Methods

The study sample was from the population-based SardiNIA Project, established in 2001, which investigates thousands of genetic and phenotypic traits associated with aging [9]. Briefly, this single-center survey started in 2001 and collected clinical and genetic data from a founder population living in four towns of the Lanusei valley, Italy. Approximately 62% of the eligible population aged at least 14 years accepted to participate. Thus, 6123 subjects (42.5% men) with a wide age span (14–102 years) were recruited and visited every three years.

All the participants signed an informed consent form. For the aim of this study, we used data from the survey. The study protocol was approved by the Ethics committee of Azienda Sanitaria Locale number 8 (protocol 24/Comitato Etico/02, authorization number 4737, approved on 19 June 2001)

### 2.1. Biochemical and Hormone Assays

After an overnight fast, blood samples were taken between 7 and 8 a.m. and stored at −80 °C until use. Plasma triglycerides and total cholesterol were determined by an enzymatic method (Abbott Laboratories ABA-200 ATC Biochromatic Analyzer, Irving, TX, USA). High-density lipoprotein (HDL) cholesterol was determined by dextran sulfate–magnesium precipitation. Low-density lipoprotein (LDL) cholesterol was calculated by the Friedewald formula. Fasting plasma glucose concentration was measured by the glucose oxidase method (Beckman Instruments Inc., Fullerton, CA, USA). The method used to assess TSH was a solid-phase, two-site chemiluminescent immunometric assay (Siemens TSH assay Immulite 2000, normal range 0.4–4.0 μIU/mL). The method used to assess FT4 was a solid-phase, enzyme-labeled chemiluminescent competitive immunoassay (Siemens FT4 assay Immulite 2000, normal range 0.89–1.76 ng/dL).

### 2.2. ABPM

ABPM was assessed with an oscillometric device (Spacelabs 90207, SpaceLabs Inc., Wokingham, Berkshire, UK). Blood pressure was automatically collected every 15 min throughout the day (7:00 a.m. to 10:00 p.m.), and every 30 min during the night (10:00 p.m. to 7:00 a.m.). Total 24 h ABPM recording of each subject lasted a minimum of 20 h.

### 2.3. Definition of Cardiovascular Risk Factors

We defined arterial hypertension the presence of either: (a) SBP ≥ 140 mmHg: (b) DBP ≥ 90 mmHg; (c) self-reported use of antihypertensive drugs. We defined diabetes mellitus as the presence of either: (a) self-reported diagnosis of diabetes; (b) use of antidiabetic drugs; (c) elevated fasting glycated hemoglobin; or (d) fasting glycaemia. Subjects were considered affected by dyslipidemia in case of self-reported use of lipid-lowering medications and/or increased LDL cholesterol levels and triglycerides (respectively, ≥140 mg/dL and ≥150 mg/dL). Smokers were defined as current consumers of at least one cigarette per day. Cardiovascular events were defined as a documented history of myocardial infarction or stroke. Menopause was considered in cases of self-reported diagnosis or age ≥ 55 years in case of missing data.

### 2.4. Statistics

Continuous data are presented as a median and interquartile range due to the skewed distribution. Categorical variables were reported as absolute numbers and percentages.

Continuous variables were compared by the Wilcoxon rank-sum test. Differences in frequencies were tested by the exact Fisher test. We firstly tested the correlation (Wilcoxon rank-sum test and Spearman’s correlation) between ABPM parameters and the main variables cardiovascular risk factors (age, BMI, total cholesterol, LDL, HDL, triglycerides, smoking, and the presence of diabetes, hypertension, dyslipidemia, and CV events), thyroid hormones (TSH and FT4) and the presence of menopause in females.

On the basis of univariate analysis, multiple linear regression tests were performed separately in males and females, with each parameter of ABPM as a dependent variable, and age, heart rate, TSH, total cholesterol, triglycerides, diabetes, hypertension, dyslipidemia, cardiovascular event, and smoking as independent variables. Menopause was further considered as covariate in females, due to its correlation with cardiovascular risk factors and thyroid disorders [10]. Collinearity was reported in cases of tolerance less than 0.1 and variation-inflation factor (VIF) greater than 10. Significance was set at *p* < 0.05 in Stata v12.0.

## 3. Results

The whole sample consisted of 3277 subjects. As reported in Table 1, males were older and had higher BMIs compared to females (*p* < 0.010). Males also had higher SBP and DBP values (*p* < 0.001 for all) and had a worse lipid profile: higher LDL (*p* < 0.01) and triglycerides (*p* < 0.001). On the contrary, females had higher HDL (*p* < 0.001) but had a higher frequency of dyslipidemia (*p* < 0.001). On the contrary, the frequency of diabetes and hypertension was higher in males (*p* < 0.001). As for thyroid function, levels of TSH were higher in females (*p* < 0.001), while FT4 was comparable between females and males who were more frequent smokers (*p* < 0.001) and had a higher frequency of cardiovascular disease.

Table 2 shows the summary of parameters in ABPM stratified by gender. The average 24 h SBP, DBP, and MAP were higher in males compared to females (respectively, 123.8 vs. 116.9 mmHg, 72.9 vs. 75.6 mmHg, and 88.3 vs. 92.5 mmHg, *p* < 0.001 for all). Similarly, daytime and nighttime SBP, DBP, and MAP were higher in males compared to females (*p* < 0.001 for all). Females had higher HR on average, over 24 h, daytime and nighttime (*p* < 0.001).

**Figure 1:**
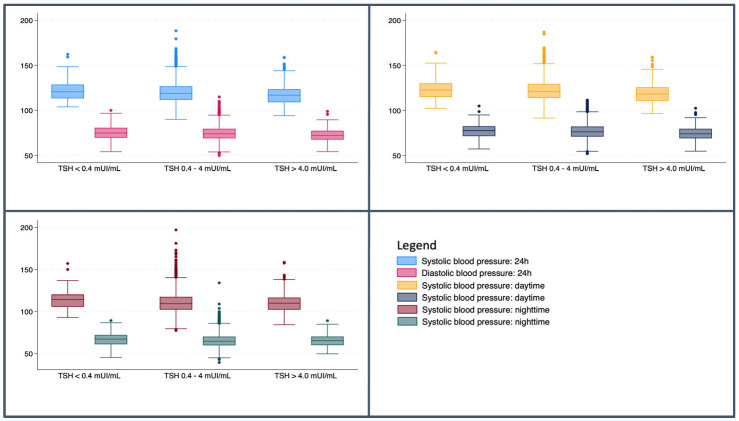
Mean systolic and diastolic blood pressure stratified by TSH values.

Figure 1 shows the mean SBP and DBP recorded by ABPM across the thyroid function. Specifically, it compares the assessment of SBD and DBP over 24 h, daytime, and nighttime accordingly to three different TSH ranges: subjects with reduced TSH, those with TSH within the reference range, and participants with TSH above the upper limit of the reference range.

Table 3 shows the spearman correlation analysis between TSH and ABPM parameters stratified by sex. TSH was negatively associated with the average 24 h and daytime SBP (respectively, rho −0.061, *p* = 0.004 and rho −0.066, *p* = 0.002), and positively associated with nighttime DBP in females (rho 0.029, *p* = 0.001). In males, TSH had a negative correlation with 24 h SBP and DPB (rho −0.070, *p* = 0.008 and rho −0.055, *p* = 0.033, respectively), daytime SBP (rho −0.080, *p* = 0.002), and daytime DBP (rho −0.060, *p* = 0.021).

Table 4 shows the results of multiple regression analyses in females. After adjusting for confounders, neither SBP nor DBP was associated with TSH. Table 5 shows the results of regression analysis in males. When considering the average 24 h and daytime, TSH was negatively associated with DBP (respectively, Coeff. −0.192, *p* = 0.025 and Coeff. −0.021, *p* = 0.018). DBP during nighttime, as well as SBP (average 24, daytime and nighttime), were not associated with variation in TSH.

## 4. Discussion

In this study, we examined associations of thyroid-related hormones with specific parameters of ABPM. We found an association between TSH with the average 24 h and daytime DBP in males, but not in females. The association between arterial hypertension and thyroid function has been documented by different studies, but results are not clear. Volzke et al. reported that subclinical hyperthyroidism was not associated with changes in blood pressure, pulse pressure, or incident hypertension [11]. In addition, the same group showed that subclinical hypothyroidism was associated with an increased risk of hypertension in children and adolescents [12]. Another study, which enrolled over 13,000 subjects, reported that hypertension was associated with subclinical hypothyroidism, particularly in females, and there was no association with subclinical hyperthyroidism [13]. The sex-specific pattern has also been evaluated by another study, a sub-cohort of the Hispanic Community Health Study/Study of Latinos [14]. Indeed, authors reported that TSH and TSH/FT4 ratios were associated with DBP: in men, a 1-SD increase in TSH and TSH/FT4 ratio was positively associated with the development of hypertension, while in women, the TSH/FT4 ratio was protective. Results from the ELSA-Brazil study showed that FT4 within the first quintile was a mild risk factor for incident hypertension in euthyroid patients and in those with subclinical dysfunction [15].

Previous studies also evaluated the association between thyroid function and blood pressure assessed by ABPM with different results. Cai et al. showed that the association between thyroid function and blood pressure changed along with the type of measurement. Indeed, the authors found that patients with subclinical hypothyroidism had comparable in-office blood pressure values, but ABPM daytime and nighttime SBP, and 24 h SBP and DBP was higher in patients with subclinical hypothyroidism [16]. Kwon et al. reported that high–normal TSH was associated with SBP in ABPM of treatment-naïve hypertensive males, but not females [17]. Another study showed that the average 24 h blood pressure was similar between hyperthyroid and euthyroid subjects [18]. Iglesias et al. found that SBP was higher in patients with overt hyperthyroidism and decreased after normalization of thyroid hormone levels [19]. The possible positive effect of the treatment of subclinical disorders is less studied, and results were not consistent. A study in nonpregnant women showed that levothyroxine treatment for 18 months showed a reduction in both SBP and DBP, in addition to the normalization of other cardiovascular risk factors [20]. The study by Zhou et al. showed that pregnant women with subclinical hypothyroidism had higher frequency of hypertensive disorders and levothyroxine treatment reduced maternal and neonatal pregnancy outcomes [21]. However, a recent systematic review and meta-analysis showed that after levothyroxine therapy, the incidences of gestational hypertension were comparable to untreated women [22].

The association between thyroid function and blood pressure could also be extended to euthyroid subjects. Indeed, a longitudinal study by Abdi et al. reported that FT4 within the reference range was directly correlated with elevated clinic blood pressure when compared to TSH, with a clear gender effect [23]. Further, the authors also showed that subjects with FT4 > 1 ng/dL were associated with increased incident pre-hypertension. Another study reported the association between thyroid function and specific subtypes of hypertension in 1056 euthyroid individuals with arterial hypertension [24]. The authors found that TSH was significantly higher in patients with sustained hypertension, while the FT4 level was higher in subjects with coat syndrome.

Our analysis showed that the association between TSH and DBP was significant only in the daytime, when compared to blood pressure assessment during nighttime. To the best of our knowledge, this is the first analysis that reported this finding, which deserves specific additional studies. Indeed, it is well acknowledged that during nighttime, the dipping phenomenon caused a reduction in blood pressure in an average of 50% of the subjects and was due to a decrease in heart rate which, in turn, decreases the cardiac output during the night. A non-dipping pattern could be due to a lesser nocturnal decline in cardiac output or to an exaggerated increase in systemic vascular resistance, which usually does not change. The measurement of TSH is the most accepted test to diagnose primary thyroid disorders. TSH has a circadian rhythm: it increases during nighttime and reaches a nadir before 8:00 p.m. regardless of gender. Conversely, tetraiodothyronine was higher in the daytime in females, while at the same time, males had lower levels [24]. While circulating FT4 is derived from the secretion by the thyroid gland, which produces only 20% of circulating free triiodothyronine, the remaining 80% is generated by the peripheral conversion of tetraiodothyronine. Triiodothyronine is considered the active form of thyroid hormone and shows a specific circadian pattern of secretion, which is delayed but parallels TSH levels [25]. The triiodothyronine effects are mediated by nuclear receptors, whose action takes at least 0.5–2.0 h [26], and therefore, the effect of the increase in triiodothyronine is only significant later in the day. The sympathetic activity may play an additional role. Indeed, a recent study showed that TSH suppressive therapy with levothyroxine increased the sympathetic activity, which, in turn, may increase vascular resistance [27]. We acknowledged that our sample has not been tested for triiodothyronine, thus impairing the interpretation of our results. In addition, the cross-sectional design of our analysis does not allow us to study the cause-and-effect relationship.

## 5. Conclusions

Daytime and daily average DBP in males were inversely related to TSH, suggesting that an excess of thyroid hormone may have a detrimental effect on the cardiovascular system. The lack of correlation during nighttime may reflect the circadian secretion of TSH and the delayed effect of thyroid hormones. Additional studies will be needed to test this hypothesis.

## Figures and Tables

**Table 1:** Summary characteristics of the sample.

Variable	Female	Male	*p* Value	Total
*n*	1862	1415		3277
Age, yrs	48.3 (36.4–61.0)	51.8 (39.8–65.2)	<0.001	49.7 (38.0–63.0)
BMI, Kg/m^2^	24.2 (21.4–27.8)	26.9 (24.5–29.6)	<0.001	25.6 (22.6–28.8)
TSH, uUI/mL	1.68 (1.15–2.47)	1.39 (0.96–1.99)	<0.001	1.53 (1.06–2.28)
FT4, ng/dL	1.07 (0.97–1.23)	1.08 (0.98–1.23)	0.216	1.08 (0.97–1.23)
SBP, mmHg	118 (109–131)	128 (119–140)	<0.001	123 (112–136)
DBP, mmHg	74 (69–80)	79 (73–87)	<0.001	76 (70–83)
Total cholesterol, mg/dL	216 (190–243)	216 (187–241)	0.266	216 (189–242)
LDL, mg/dL	133 (111–157)	138 (114–162)	0.007	135 (112–158)
HDL, mg/dL	60 (52–69)	49 (42–57)	<0.001	55 (47–65)
Triglycerides, mg/dL	88 (65–122)	111 (81–160)	<0.001	97 (71–137)
Diabetes, n (%)	95 (5.1%)	118 (8.3)	<0.001	213 (6.5%)
Hypertension, n (%)	443 (23.8%)	556 (39.3%)	<0.001	999 (30.5%)
Dyslipidemia, n (%)	520 (27.9%)	476 (33.6%)	<0.001	996 (30.4%)
CV event, n (%)	29 (1.6%)	49 (3.5%)	<0.001	78 (2.4%)
Smoke, n (%)	254 (13.6%)	320 (22.6%)	<0.001	574 (17.5%)
Menopause, n (%)	858 (46.1%)	-	-	-

Abbreviations: BMI, body mass index; TSH, thyrotropin; FT4, free thyroxine; SBP, systolic blood pressure; DBP, diastolic blood pressure; LDL, low density lipoprotein; HDL, high density lipoprotein; CV, cardiovascular. Wilcoxon rank-sum test was used to compare by gender all continuous variables and differences in frequencies were tested by the exact Fisher test.

**Table 2:** Parameters in ambulatory blood pressure monitoring by gender.

ABPM parameter	Female	Male	*p* Value	Total
24 h				
SBP, mmHg	116.9 ± 11	123.8 ± 11.3	<0.001	119.9 ± 11.6
DBP, mmHg	72.9 ± 7.41	76.5 ± 8.05	<0.001	74.5 ± 7.91
MAP, mmHg	88.3 ± 8.12	92.5 ± 8.53	<0.001	90.1 ± 8.56
HR, bpm	77.3 ± 8.51	73.0 ± 8.69	<0.001	75.5 ± 8.85
Daytime				
SBP, mmHg	119.1 ± 11.3	126.1 ± 11.5	<0.001	122.1 ± 11.9
DBP, mmHg	75.1 ± 7.71	78.8 ± 8.4	<0.001	76.7 ± 8.23
MAP, mmHg	90.5 ± 8.41	94.8 ± 8.85	<0.001	92.4 ± 8.86
HR, bpm	79.4 ± 8.9	74.9 ± 9.13	<0.001	77.5 ± 9.28
Nighttime				
SBP, mmHg	107.5 ± 14.9	113.5 ± 16.0	<0.001	110.1 ± 15.7
DBP, mmHg	63.6 ± 9.38	67.0 ± 10.6	<0.001	65.1 ± 10.1
MAP, mmHg	78.8 ± 10.9	82.8 ± 12.0	<0.001	80.6 ± 11.6
HR, bpm	68.5 ± 10.5	65.3 ± 10.7	<0.001	67.1 ± 10.7

Abbreviation: ABPM, Ambulatory blood pressure monitoring; SBP, systolic blood pressure; DBP, diastolic blood pressure; MAP, mean arterial pressure; HR, heart rate. Wilcoxon rank-sum test was used to compare ABPM parameters by gender.

**Table 3:** Spearman correlation between TSH and ABPM parameters.

ABPM parameter	Females		Males	
	rho	*p* Value	rho	*p* Value
24 h *				
SBP	−0.061	0.004	−0.070	0.008
DBP	−0.016	0.454	−0.055	0.033
Daytime *				
SBP	−0.066	0.002	−0.080	0.002
DBP	−0.026	0.232	−0.060	0.021
Nighttime *				
SBP	−0.036	0.800	0.022	0.401
DBP	0.029	0.001	0.030	0.254

Abbreviation: ABPM, Ambulatory blood pressure monitoring; SBP, systolic blood pressure; DBP, diastolic blood pressure. * Models adjusted for age, heart rate, BMI, total cholesterol, triglycerides, diabetes, hypertension, dyslipidemia, cardiovascular event, smoking, and menopause.

**Table 4:** Correlation between TSH and ABPM parameters in females at multivariate analysis.

Dependent Variable	Coeff	s.e.	t	*p* Value
24 h *				
SBP	−0.041	0.17	−0.23	0.816
DBP	−0.073	0.13	−0.58	0.559
Daytime *				
SBP	−0.113	0.18	−0.63	0.527
DBP	−0.140	0.13	−1.08	0.281
Nighttime *				
SBP	0.188	0.19	0.96	0.338
DBP	0.197	0.13	1.47	0.141

Abbreviation: Coeff, beta coefficients; s.e., standard errors of the coefficients; t, t-test; SBP, systolic blood pressure; DBP, diastolic blood pressure. * Models adjusted for age, heart rate, BMI, total cholesterol, triglycerides, diabetes, hypertension, dyslipidemia, cardiovascular event, smoking, and menopause.

**Table 5:** Correlation between TSH and ABPM parameters in males at multivariate analysis.

Dependent Variable	Coeff	s.e.	t	*p* Value
24 h *				
SBP	−0.141	0.12	−1.19	0.234
DBP	−0.192	0.09	−2.24	0.025
Daytime *				
SBP	−0.166	0.12	−1.36	0.173
DBP	−0.021	0.09	−2.36	0.018
Nighttime *				
SBP	−0.069	0.13	−0.52	0.601
DBP	−0.142	1.00	−1.49	0.137

Abbreviation: Coeff, beta coefficients; s.e., standard errors of the coefficients; t, t-test; SBP, systolic blood pressure; DBP, diastolic blood pressure. * Models adjusted for age, heart rate, BMI, total cholesterol, triglycerides, diabetes, hypertension, dyslipidemia, cardiovascular events, and smoking.

## Data Availability

The data presented in this study are available on request from the corresponding author. The data are not publicly available due to privacy.
